# Cognitive Control of Escape Behaviour

**DOI:** 10.1016/j.tics.2019.01.012

**Published:** 2019-04

**Authors:** Dominic A. Evans, A. Vanessa Stempel, Ruben Vale, Tiago Branco

**Affiliations:** 1 Sainsbury Wellcome Centre for Neural Circuits and Behaviour, UCL, London, UK; 2 These authors contributed equally to this work

**Keywords:** instinctive decisions, defence, threat, behavioural flexibility

## Abstract

When faced with potential predators, animals instinctively decide whether there is a threat they should escape from, and also when, how, and where to take evasive action. While escape is often viewed in classical ethology as an action that is released upon presentation of specific stimuli, successful and adaptive escape behaviour relies on integrating information from sensory systems, stored knowledge, and internal states. From a neuroscience perspective, escape is an incredibly rich model that provides opportunities for investigating processes such as perceptual and value-based decision-making, or action selection, in an ethological setting. We review recent research from laboratory and field studies that explore, at the behavioural and mechanistic levels, how elements from multiple information streams are integrated to generate flexible escape behaviour.

## Highlights

Escape behaviours are not only simple stimulus-reactions but are under cognitive control, allowing the study of processes such as decision-making and action selection in tractable organisms in ethological settings.

Successful escape relies on integrating multiple external and internal variables, such as for computing flight trajectories towards shelter, and implementing trade-offs by choosing between actions that satisfy competing motivations.

Some neural mechanisms of escape are innate and conserved across species, but are subject to control and modification by multiple systems, including the neocortex, which allow experience to be flexibly incorporated into escape behaviour.

New tools to quantify behaviour while recording neural activity enable analysis of ethologically-relevant behaviours in complex environments, and will advance our understanding of the neural basis of natural behaviours.

## A Wide Range of Complexity in Escape Behaviours

**Escape** (see Glossary) is an **instinctive defensive behaviour** that has evolved to avoid harm from predators and other threats in the environment. Animals that fail to escape from imminent threats will suffer reduced fitness, catastrophically in the case of death, but also in the case of injury, for example owing to reduced ability to forage for food or weakening in social status [1]. Evolution has produced many different expressions of escape behaviour that reflect aspects such as biomechanics, the nature of the threat, local ecology or individual history, and which range from simple to extraordinarily complex [2]. At one end of the spectrum, animals might escape a fast-approaching predator by moving away with reflex-like actions, such as the ‘jack-knife’ tail flip in crayfish and the **C-start** escape in fish 3, 4. At the other end, successfully escaping from threats can require cognitive processes, including using memory and deciding between alternative options. For example, animals escaping in complex environments need to use knowledge of refuge locations and escape routes, and prey escaping from a pursuing pack of predators must dynamically compute escape strategies and trajectories 5, 6. In addition, one of the most important components of escape behaviour are economic trade-offs because escaping from a foraging patch incurs a potential loss of resources that escape decisions should take into account for optimizing fitness in the long term 7, 8. The computation of escape behaviour therefore integrates information from various streams, which creates the flexibility necessary for animals to survive in dynamic environments, and produce escapes that minimize reaction time in response to imminent threats, or that maximize success by considering as much information as possible. This information can be extracted at the time of the encounter (e.g., the nature of the threat and the current state of the environment), derive from prior experience (e.g., expectation about the outcome of the escape action), and also arise from internal signals of the state of the animal, such as hunger or anxiety.

**Figure 1:**
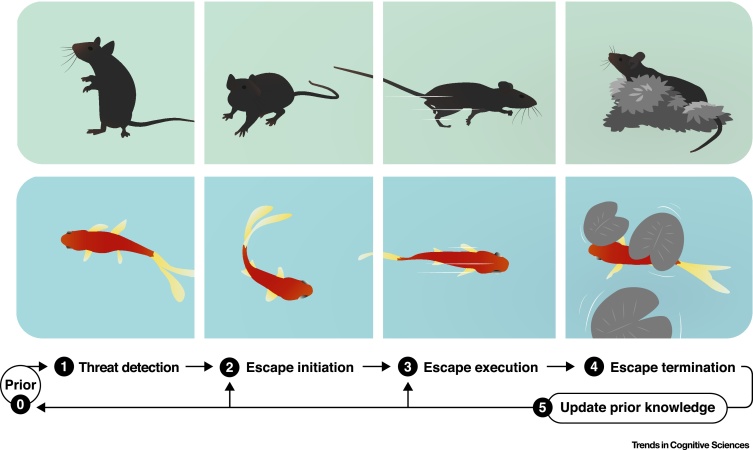
A Conceptual Timeline of Events during Escape Behaviour. Illustrations of a mouse (top) and fish (bottom) at different time points during an escape response away from a predator and towards a refuge (from left to right). **Prior knowledge (0)**: learning experiences, such as previous encounters with predators, influence escape behaviour. This prior knowledge is updated constantly throughout the life of the animal **(5)** and can modulate each part of the escape sequence. **Threat detection (1)**: the animal detects a sensory stimulus and must evaluate whether it is a potential predatory threat. This can be done through specialised innate detection pathways or learning processes, and includes behaviours that facilitate threat assessment, such as freezing. **Escape initiation (2)**: once a stimulus is considered to be threatening, the decision and timing of escape depend on trade-offs such as the presence of nearby desirable resources, and variables such as the availability and distance to shelter. **Escape execution (3)**: in environments that are spatially simple, animals accelerate and flee away from the threat, and often in a straight trajectory towards shelter. Escape is however a dynamic process that can take into account properties of the threat and of the local surroundings. **Escape termination (4)**: the escape action terminates when the animal has reached safety, either by increasing the distance from the threat source or by arriving at the shelter location.

**Figure 2:**
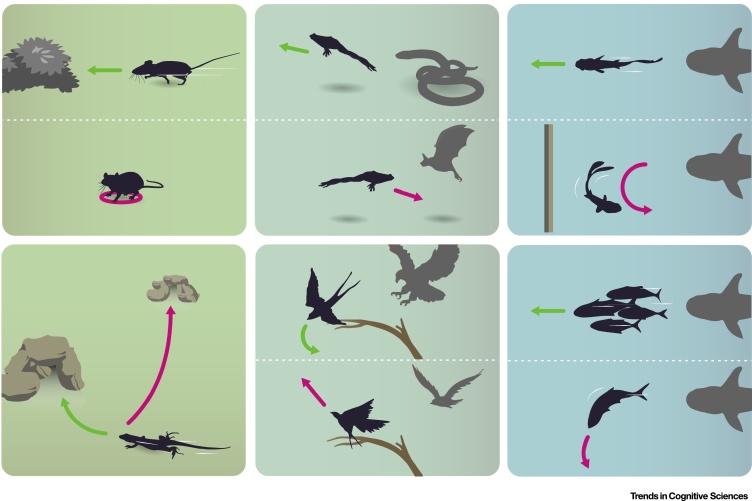
Flexibility of Escape Execution in Different Species.**Escape directionality depends on the presence and location of shelter** (left panels). (Top) When an animal has knowledge that a refuge is inaccessible, absent, or too far away, the predominant response to threat switches from escape to freezing behaviour. (Bottom) The presence of a suitable refuge in the environment guides precise escape trajectories to its location. If a refuge becomes unavailable and the animal finds a new one, flight paths are modified accordingly very rapidly. **Escape trajectories are threat-dependent** (middle panels). (Top) The type and location of a predator influences escape trajectory. A frog directs its escape away from a terrestrial predator such as a snake, but flees towards an aerial predator such as a bat to undercut their flight path [117]. (Bottom) Animals including birds, deer, fish, and frogs flee directly away from threatening stimuli, which may function to maximize the distance between predator and prey. The same animals can also escape at a 130–90° angle, for example, to facilitate visual monitoring of predators during escape in response to less threatening stimuli, or as a less predictable, evasive manoeuvre in response to a fast predatory strike [118]. **The physical and social environment modulates escape** (right panels). (Top) Flight trajectories take into account the presence of obstacles in the environment. For example, fish that usually flee away from an approaching predator may escape towards it if an obstacle occludes the optimal escape path. (Bottom) Solitary fish can initiate escapes at various onset angles, whereas schooling fish escape in straight and uniform trajectories owing to the spatial constraints imposed by the shoal.

The ability to robustly trigger escape in a stimulus-dependent manner, together with the potential for investigating it in conditions that require different levels of cognitive complexity, make escape behaviour a powerful ethological model for systems neuroscience and mechanistic studies of cognition. Classic ethological field work has revealed many principles of escape at the behavioural level [5], whereas laboratory studies aiming to understand the neurophysiology of escape have traditionally focused on fast stimulus-escape reactions, such as jumping escapes in locusts and flies 9, 10. There is thus a gap between our understanding of the biological mechanisms of escape and the complex behaviours displayed by animals escaping in natural environments. However, recent advances in behaviour monitoring and recording techniques in freely moving animals promise to bridge this gap, and open the way for understanding how neural circuits implement the cognitive processes that control escape behaviour. Here we review evidence that escape is a flexible behaviour under cognitive control, as well as some of the currently known underlying neural mechanisms (see further references in the supplemental information online). We consider three time points: threat detection, escape initiation, and escape execution (Figure 1). In addition to structuring escape into separate control points, this division generalizes to classes of problems that the brain must solve when computing any behaviour, namely classifying sensory information, selecting, and then executing flexible actions (Figure 2), and further underscores the power of using escape behaviour as a model for systems neuroscience.

## Threat Detection

The first step in the computation of escape behaviour is to evaluate sensory information to identify whether a threat is present. This is a classification operation where sensory stimuli are sorted into threatening or not-threatening, and includes a perceptual component (is the stimulus there?) and a value component (is it threatening or not?). Animals must detect, identify, and evaluate threats based on sensory features such as shape, size, speed, or smell. For most animal species there are stimuli that innately carry negative valence, which is essential for surviving and escaping from predators without needing to rely on prior exposure to learn that they are threatening. This can be implemented through the evolution of dedicated channels with specialised sensory detectors, which activate defensive circuits and lead to stereotyped behaviour. For example, the rodent olfactory system has a circuit specialised for the detection of chemical substances emitted by other species, **kairomones**, where the receptors are located in the vomeronasal organ and connected to the hypothalamic defensive system via the amygdala [11]. Similarly, a subset of cells in the visual system increase their firing rate in response to dark expanding stimuli, which signal approaching objects on a collision course and elicit defensive responses without prior experience [12], even in human infants [13]. These cells can be found across the animal kingdom 14, 15, 16, 17, probably reflecting the strong selective pressure for a collision-avoidance system. In vertebrate species, the optic tectum (OT) and its mammalian homologue, the superior colliculus (SC), are conserved regions in the midbrain for detecting and integrating threats 18, 19, 20. While the role in defensive behaviour is perhaps the least well understood for the SC in mammals, neural activity recordings have revealed **looming**-sensitive cells 16, 17, 19, 21, 22, 23, and activity manipulations can both impair or elicit defensive behaviours 19, 23, 24, 25, 26. For innately threatening sensory stimuli that are simple and unimodal, the microcircuitry and computational mechanisms of threat integration in the OT/SC are starting to be uncovered 19, 27, 28, 29, 30, and place these midbrain circuits at the core of threat processing.

### Previous Predator Encounters Modify Threat Perception and Escape

Although reflex-like stimulus–response couplings are important for survival, the evaluation of threats should be flexible enough to adapt to dynamic contingencies, and be modulated by expectations arising from experience and prior beliefs. For example, prey should adjust their behaviour based on the frequency and outcome of past encounters with predators, and adapt to the current level of predatory risk. In theoretical terms this is equivalent to a prior – at high predation risk, the threshold for detecting or reacting to threats should be lowered, and raised for low predation risk. Such flexible adaptation has been demonstrated in laboratory and field studies, where animals quickly learn to suppress escape responses if they are repeatedly presented with **innately threatening stimuli** but no adverse outcome occurs, presumably to minimise costs associated with unnecessary escape responses [31]. In animal studies, the most common way to determine whether a stimulus is threatening is to observe behaviour, and therefore it is sometimes not possible to distinguish whether adaptations take place at the stimulus classification stage or at the point of action selection (see below). The process of habituation to a threatening stimulus can be strongly context-dependent [32] and stimulus-specific 33, 34, whereas the time course of its acquisition and persistence is variable across stimuli, contexts, and species 34, 35. This high level of flexibility suggests that there is no single underlying mechanism for escape habituation, but rather, that it is composed of multiple distinct processes operating at different levels in the same animal 36, 37, 38. Although the neural mechanisms of escape-behaviour adaptations are mostly unknown, they have been suggested to arise from changes in inhibitory or neuromodulatory tone in crustaceans 37, 39. It will be interesting to consider whether behavioural habituation to innate threats is purely non-associative or whether it can also be shaped by associative processes, similar to inhibitory learning during conditioned fear extinction [40]. In mammals, threat-detection circuit elements such as the SC receive both top-down cortical as well as strong neuromodulatory input 21, 41, 42, 43, and are therefore well-positioned to implement escape habituation through cognitive control.

Across species, experience with threats can also lead to heightened sensitivity to future stimuli. Many animals respond to repeated approaches from predatory threats by increasing the probability of escape and **escape vigour** in subsequent encounters 44, 45, decreasing escape reaction times [46], and changing tactics [47]. These changes in escape performance are thought, at least in part, to be under cognitive control 39, 48, and appear to adapt escape behaviour to higher levels of predation risk [49]. The detection of predatory stimuli in one sensory modality can also cause sensitization in others, as evidenced by the increase in behavioural and olfactory neuron sensitivity observed in moths after brief exposure to bat calls [50]. Classically, such generalization of sensitization is related to a global state of increased arousal and stress 51, 52. In lizards, for example, sensitisation is correlated to increased corticosterone levels, which regulate stress responses, and blocking corticosteroid elevation renders lizards unable to adapt their reaction in response to repeated predator exposure [53]. In mice, stress exposure has been shown not only to decrease the response latency to looming visual stimuli [54] but also to increase the duration of freezing [55]. It will be interesting to understand which mechanisms of escape sensitisation are specifically cognitive, such as faster categorization of predatory threats through learning, and which arise from global mechanisms such as stress.

Habituation and sensitisation are two opposite adaptation strategies, and whether an individual responds to repeated threat encounters with one or the other is likely to depend on factors such as the initial perceived threat-level of the stimulus and the frequency of encounter, and in fact both can occur in the same animal on different timescales 56, 57.

### Associative Learning Expands the Ability To Detect Threats

In addition to updating innate processes for classifying sensory stimuli as threats, surviving encounters with predators should also lead to learning new associations between features of the sensory space that might predict future encounters [8]. This has been extensively investigated in the laboratory, where associative learning can be easily triggered by pairing a noxious stimulus such as a foot shock with a neutral stimulus. These Pavlovian threat conditioning protocols usually trigger freezing, and have been one of the main paradigms for studies of fear learning mechanisms 58, 59, 60. In more naturalistic scenarios, relying on the experience of pain to learn new associations would be a dangerous strategy, and animals are also capable of performing such associations by computing temporal and contextual coincidence between innately threatening stimuli and neutral cues. For example, cyprinid fish detect an innate danger signal released from injured conspecifics, which they can associate with sensory cues of novel predators [61]. Interestingly, prey can generalize their acquired predator recognition to similar but novel species, and continuously update their recognition templates 62, 63. Animals also learn to avoid locations associated with predation: ants are able to form a generalized memory of their predators’ pit traps after escaping a single time [64], and mice show risk assessment and escape behaviours when exploring an arena in which they previously encountered threats 19, 65.

In mammals, the circuits for encoding predator cues and predator context-specific memories are not as well understood as those that process simple aversive stimuli such as foot shocks. Overall, they seem to involve the same core associative-learning circuit elements of the basolateral amygdala, hippocampus, and distributed cortical regions, but with the additional integration of threat-instructive signals from networks such as the medial hypothalamic defensive system [66]. It will be particularly interesting to understand how learned associations feed into innate threat-detection and escape decision centres, such as the midbrain, to inform escape and other defensive behaviours.

### Vigilance and Active Risk Assessment Enhance Threat Detection

In addition to modulation from past experience, threat detection is also affected by the current state of the environment, and foraging animals often display threat-assessment behaviours [67] that are flexibly adapted to the current environmental conditions, to improve risk analysis. For example, birds that engage in sentinel behaviour to protect foragers from predators, such as pied babblers, start epochs of guarding sooner and for longer periods in higher grass (reduced visibility) and in high wind (reduced audibility), during which they adopt a raised position to scan for danger and communicate alarm signals [68]. Wallabies are similarly more vigilant at higher wind speeds [69], indicating that decreased availability of auditory, visual, and olfactory cues is actively compensated for by an increase of attention. This allocation of resources to threat detection, such as attention and vigilance, emphasises that threat detection is an active process. In fact, immediately upon detecting a threat, most animals interrupt ongoing behaviours and freeze – a behaviour that aids risk assessment by allowing animals to update information about the local environment, for example to compute the likelihood of threat presence, and to estimate and evaluate the threat risk in comparison to the benefits of not fleeing (e.g., proximity to a food source) 70, 71, 72.

It is interesting to consider that key areas for threat detection and expression of defensive behaviours, such as the SC and amygdala, are also involved in the control of spatial attention and saliency maps 73, 74, 75, 76. This may suggest that threat detection is fundamentally a process similar to others that link salient events in sensory space with actions – for threat and escape this link may be innate, and modified through experience.

## Escape Initiation

After a sensory stimulus has been detected and valued as being a threat that requires immediate action, the escape circuit should in principle be engaged as soon as possible, and evolution has shaped neural circuits to ensure that this can be achieved. The Mauthner cell is perhaps the best-studied example – it receives direct synaptic input from the cranial nerve VIII carrying information from hair cells, and commands the initiation of fast C-start escape responses by projecting across the midline to activate contralateral motoneurons, allowing millisecond-long latencies between threat detection and escape start [3]. In mice, the periaqueductal gray (PAG) circuit that controls escape initiation 19, 24, 25, 26, 77 receives monosynaptic input from the SC [19], which itself receives direct retinal input [78], thereby providing a short pathway between threat detection centres and escape initiation. However, escape initiation is often probabilistic and modulated by many variables. It has long been recognised that animals do not necessarily flee immediately once predators are detected, a point stressed by Ydenberg and Dill [7], who disputed much of the previous literature that equated escape initiation with threat detection. There are several processes that can account for the variable time period between detecting a threat and starting to escape, and which also control whether escape happens at all.

### Decision Processes and Economics Control the Onset of Escape

Initiating escape can be the result of a decision-making process that requires integrating dynamic evidence about the threat, and decisions take time. Therefore, the escape reaction time should in part reflect the time over which decision-making processes occur, such as evidence accumulation. It is important to note, however, that even in this case reaction time does not necessarily reflect exclusively the time it takes to decide to escape: animals may have made a commitment to escape, but not initiated the action, such as during ambush encounters with a striking predator, where an animal waits to take evasive action until it is too late for the predator to adjust its trajectory. In addition, economic models of escape, supported by experimental evidence from field studies, suggest that animals escape only when the costs of remaining (e.g., the level of risk of injury or predation) are higher than the costs of fleeing (e.g., loss of foraging or mating opportunities) 5, 7. An important variable in this computation is the internal state of the animal because it dictates the value of actions that compete with escape. Behavioural studies have shown a tight link between animals’ internal state − such as hunger or the receptive period of the sexual cycle – and their escape strategies 79, 80. Complementary studies on laboratory animals have begun to produce a detailed understanding of the circuits and mechanisms by which internal states and motivations control defensive behaviours (Box 1 for details on hunger) 54, 81, 82. Current efforts to map motivational states onto distinct neuronal pathways and cell types will enable us to determine how different behavioural motivations compete, and shed light on how they act mechanistically to influence defensive decision-making.

Box 1 **Hunger’s Hold on Escape**

Hunger has a powerful and well demonstrated effect on behavioural choices and learning [119]. For example, animals show a preference for food items that they have previously encountered in a hunger state over items that they ate while sated, even if the energetic value of both food items is the same [120]. Defensive behaviours are no exception to the control of behavioural decisions by hunger, which has been shown to directly modulate escape in several species. Crayfish, for example, reduce the frequency of tail-flip escapes in response to overhead sweeping visual stimuli when they are fasted [79]. In larval zebrafish, recent work has uncovered neural mechanisms that can explain how hunger decreases escape probability via changes in the visual processing of approaching stimuli in the optic tectum. In these animals, food deprivation inhibits the hypothalamic–pituitary–interrenal axis and increases the activity of serotonergic neurons in the raphe: this serotonergic input to the tectum then recruits cells that are tuned to small stimuli, causing an over-representation of food-like objects, which has been suggested to bias the choice to approach over escape 121, 122. Mice also display more risk-seeking behaviours when hungry, including spending more time in threatening environments [81]. Recent laboratory studies have dissected the neural circuits for hunger in rodents 123, 124, and have shed light on the nodes of these circuits that link hunger states to defensive behaviours. Notably, activation of a single, molecularly-defined population of inhibitory neurons in the hypothalamic arcuate nucleus, AGRP^+^ neurons, can recapitulate the effects of hunger on defence 81, 82, 119, providing an exciting entry point for understanding behavioural choices in face of competing motivations, and the neural basis of instinctive value-based decisions.

Alt-text: Box 1

Overall, the decision to initiate escape is not simply a threat but detection process, it is a computation that can integrate multiple external and internal variables, and currently we know very little about how the brain implements this process.

### Environment and Threat Properties Control Escape Selection

Escape is only one of several possible actions in the defensive repertoire of most animals, and, for each encounter with a threat, the defensive action with the highest likelihood of success should in principle be chosen. The optimal choice depends on multiple factors about both the environment and the properties of the threat, which should be taken into account when selecting escape or alternative actions. A canonical defence alternative to escape is freezing behaviour, which has the main goal of avoiding detection [67]. One of the most intriguing aspects of the selection between escape and freezing is that it is determined by knowledge of whether or not there is a shelter in the environment. This has been observed in lizards [83] and several rodent species, which can learn about shelter existence in seconds and very quickly update their defensive strategy 84, 85 (Figure 2, top left panel). In addition, in various animals including squirrels [86], the prey–predator distance that determines escape onset depends on the distance to refuge. This suggests that the escape initiation network is under the control of neural circuits that encode a spatial representation of the environment, which can gate escape initiation (but also control escape trajectories, see below). In rodents, the midbrain defence network receives direct synaptic input from many cortical areas that could convey this type of spatial information 42, 87, but it is unknown which areas are relevant and how such control might be implemented.

Another factor known to control escape selection is the presence and value of a desirable resource. For example, when crayfish are close to a food source, they prefer to freeze in response to fast-expanding looming stimuli instead of escaping with a tail-flip [88]. In this situation freezing is advantageous because a tail-flip moves the animal away from the food source and delays resumption of foraging. Another factor that can also carry information that dictates defensive action selection is the threat stimulus itself. Escape initiation from looming stimuli depends on the approaching object surpassing a critical visual angle or speed 20, 27, 89, 90, and stimuli that slowly sweep overhead, mimicking a searching bird of prey, bias the choice of strategy towards freezing [70]. In flies, a recent study has shown that walking speed controls the selection between freezing and fleeing, suggesting that the ongoing behavioural state of the animal can bias the choice of defensive behaviour [91].

In contrast to invertebrates, the neural implementation of defensive action selection in mammals is poorly understood. For learned threats in laboratory animals exposed to fear conditioning protocols, the central amygdala (CeA) is a critical node in controlling freezing behaviour through projections to the ventrolateral PAG (vlPAG) [58], and recent evidence suggests that a competing population of CeA neurons is involved in selecting defence strategies such as escape or jumping, possibly through projections to the dorsal PAG (dPAG) [59]. For responses to innate threats, which do not necessarily rely on amygdala circuits 19, 92, 93, a similar winner-takes-all mechanism for selecting between escape or freezing could be implemented directly in the PAG, for example through mutual inhibition between the dPAG and vlPAG [59]. In agreement with its crucial role in threat processing, activation of the medial SC (mSC) can evoke both escape and freezing behaviours [94], but it is not known how mSC activity is converted into one action or the other. Interestingly, a mSC projection to the lateral posterior nucleus of the thalamus appears to be important for mSC-evoked freezing responses [95], and it is possible that there are distinct threat-responding mSC cell populations that project predominantly to either the escape or freezing circuits.

## Escape Action

Once the escape action has been selected, a key consideration is where to escape to. One of the simplest actions is to increase the distance from the threat by moving away from it. Escape responses in fish again provide an excellent example of such evasive action, where C-start escapes bend the fish with the head pointing away from the stimulus, followed by a second phase of swimming away. However, the success of the escape action will increase if it is sufficiently flexible to adapt to the properties of the threat, such as different predator strategies (Box 2), and to the properties of the environment, such as the location of refuges and potential hazards.

Box 2 **The Predator’s Point of View**

For the predator, successful predation includes at least five timepoints: detection, classification, approach, subjugation, and consumption [125]. Correspondingly, prey are equipped with defence mechanisms that target termination of the interaction with the predator at each, or multiple, of these time points. Prey defensive behaviour therefore occurs within a framework set by the predator, and we believe that the predator’s perspective should be considered in studies of prey defensive decisions for two main reasons:

**(i)** As prey behavioural responses are guided by the predator's current and expected actions, understanding the predator’s behaviour can help to infer and interpret the goals, strategies, order, and kinematics of prey behaviour 126, 127. For example, analysis of aerial pursuit trajectories, incorporating data taken from predator-mounted video cameras, has demonstrated how prey can terminate pursuit by using flanking turns that briefly withdraw the prey from the visual or acoustic field of view of the predator 128, 129. Defensive strategies can be highly complex in naturalistic predator–prey encounters. This is apparent in birds and mammals that display defensive signalling behaviours, such as communicating precise predator-related information to conspecifics, or deterring attack and pursuit by communicating their knowledge of a predator’s ambush location to the predator directly 130, 131. We should therefore appreciate that assays lacking interacting predators risk underestimating the natural behavioural repertoire of the prey, or misinterpreting the functions of particular behaviours.

**(ii)** Many prey act as predators themselves, and evidence suggests that the computations for detection, sensorimotor transformations, and movements that underlie predation and evasion are sufficiently similar that both can be carried out by overlapping neural structures. For foraging animals, sensory cues that are very similar can arise from prey, predators, or harmless agents, and misidentification can carry considerable fitness costs [132]. In the laboratory, visual cues such as small spots can elicit either approach or escape behaviour in zebrafish, frogs, and crabs, depending on their size and location in the visual field 122, 133, 134, 135, while mice will approach and capture crickets, but freeze to similarly sized overhead visual stimuli 70, 136. In zebrafish, visual processing of prey-like stimuli has been localised to specific pretectal and tectal regions 121, 122, 133, 134, 137, suggesting at least partial circuit overlap in predator and prey detection and classification processes.

Interestingly, despite their opposing goals, some of the movements required for predation and evasion are so similar [138] that prey-capture behaviour of goldfish incorporates Mauthner-mediated C-starts [139], and it has been suggested that archer fish use Mauthner cells in predictive prey-capture turns [140]. In rodents, the SC is involved in approach and prey-capture behaviours as well as defence. However, activity manipulations and lesions implicate the lateral subregion of the SC in approach and prey capture, whereas the medial subregion is more strongly implicated in defence and, interestingly, the two subregions possess partially segregated input and output connections in rodents 17, 42, 94, 141. Furthermore, several recent studies have begun to deconstruct the behavioural modules of hunting in mice by examining the role of specific projections to the PAG from the CeA, lateral hypothalamus, and medial preoptic area which can drive prey pursuit through projections to the mesenphalic locomotor region, whereas CeA to reticular formation projections evoke killing bites 142, 143, 144. How all of these network elements interact dynamically to produce the successive stages of visually-guided predation is not yet clear; however, revisiting the dual, comparative study of predation and evasion using modern techniques should be a powerful paradigm for uncovering general principles of how the brain generates goal-directed behaviour.

Alt-text: Box 2

### Properties of the Threat Determine Escape Patterns

The most basic information to extract from the threat is its type and location, which can dictate the direction of escape not only for simple behaviours but also for more complex actions (Figure 2, top middle panel). For example, flies engage in flexible visually-controlled postural adjustments approximately 200 ms before take-off to direct escape initiation away from a looming stimulus [96], and during flight they perform fast banked turns away from the stimulus that dynamically adapt to the position of the fly in relation to the stimulus [97]. Interestingly, some species introduce deliberate variability in both the initial direction of escape and the escape trajectory when moving away from a threat (Figure 2, bottom middle panel). For example, cockroaches have an unpredictable initial escape direction which, although directed away from the source of threat, falls within one of a few stereotypical directions, between 90° and 180° from the stimulus [98]. Other species make continuously unpredictable movements during escape [99] instead of optimising speed [100], which can increase survival, particularly when escaping from ballistic capture. Such unpredictability can be achieved by gait transitions, and by changes in speed and direction, as seen in the bipedal escape responses of jerboas, a hopping desert rodent [101].

Many species are capable of producing fast stereotyped escape, as well as slower and more variable responses that map onto distinct neural circuits, where the chosen type of escape depends on properties such as threat intensity and threat approach speed. In fish and amphibians this generally corresponds to eliciting a Mauthner system-mediated response 102, 103 or Mauthner system-independent escape 20, 103. Recent work in larval zebrafish has shown that escape probability and direction are modulated by the speed of a visual stimulus, where looming that mimics a fast-approaching predator elicits ‘perfect avoidance’ turns of 180° and fast reaction times, whereas slow looming leads to less predictable responses with variable bend angles and reaction times. Similar behaviour is observed in crayfish, which engage in stereotyped, giant-fibre-mediated tail flips in response to abrupt threats, and flexible, non-giant-fibre-dependent tails flips directed away from the source of aversion or towards specific locations when facing a gradual threat [104]. Flies also show threat-dependent flexibility of escape actions [105], performing either slow or fast take-offs in the early phase of escapes, thereby optimizing wing stability or speed, respectively 96, 106. This decision depends on the retinal angular velocity and size of looming stimuli, and information about each feature is conveyed to the giant-fibre escape circuit by a set of non-overlapping visual projection neurons [90]. Similarly, crabs show flexibility in escape responses to looming stimuli, adjusting the speed of escape dynamically as a function of the expansion properties of the loom [89]. Humans also seem to rely on two parallel escape circuits [48]: one for quick decisions in the face of very imminent threats, via the PAG and midcingulate cortex, and another for response to nonimminent threats, involving the posterior cingulate and ventromedial prefrontal cortices as well as the hippocampus.

Recent neurophysiological and behavioural data show comparable response flexibility in mice exposed to expanding spots of different contrast. Although it is not clear whether mice display two distinct modes of escape, flight vigour is strongly modulated by threat salience, and excitatory cells of the deep mSC (dmSC) seem to encode a variable correlated with threat-stimulus saliency, which activates the dPAG network to initiate escape once dmSC activity exceeds a threshold [19]. Activity of dPAG neurons strongly correlates with escape speed 19, 24, 77, and, as threat saliency increases, stronger activation of dmSC neurons leads to higher firing frequency in the dPAG, thereby suggesting a mechanistic link between threat-stimulus intensity and escape response vigour [19].

### Knowledge about the Environment Controls Escape Execution

A crucial determinant of escape success is taking into account the spatial features of the local environment, such as routes to shelter and the presence of obstacles, which have been shown to modulate escape responses across phyla. In fish and frogs, the onset of C-start responses is sensitive to stationary obstacles: in open water the initial C-start angle is a function of the angle of the approaching threat, but when these animals are close to a wall their escape trajectories cannot be predicted from the threat-stimulus approach angle, and are instead biased away from the wall, even if the animal needs to turn towards the approaching threat [107] (Figure 2, top right panel).

Many animals, including fish, lizards, and rodents, escape towards a known refuge [5]. Shelter-directed escapes can be a navigational challenge because the shelter might not be immediately visible from the current location, and therefore require the computation of an escape route from the current position to a previously memorised location. In agreement, rodents do not need to see the shelter nor rely on proximal visual or olfactory cues to successfully escape to it, but instead use a rapidly formed and flexible memory of the shelter location 6, 85. Intriguingly, mice terminate escape when reaching the shelter location even after it has been moved, suggesting that shelter cues are also not necessary to stop escape, and that this instead might rely on the comparison of current position with a spatial location derived from other sources [85]. In support of the notion that escape to shelter is a behaviour with the primary goal of reaching safety instead of simply moving away from threat, mice initiate escape with a head-rotation movement towards the shelter followed by an acceleration straight towards it, regardless of the initial position of the mouse and even if this means approaching the threat [85]. The selection of refuge is influenced by several variables, including the safety value of the shelter, the distance and relative position of the predator, and competition for access 108, 109, 110 (Figure 2, bottom left panel).

In addition, the local social environment can also modulate escape behaviour (Box 3). Schooling herring have uniform escape trajectories that are less flexible than the responses of solitary animals, and which decrease the likelihood of collisions [111], and even fast-response systems, such as the C-start in guppies, can exhibit similar dependency [112] (Figure 2, bottom right panel). In crayfish, social hierarchy affects the excitability of the lateral giant (LG) escape circuit in a serotonin-dependent manner [113], causing reduced LG excitability in subordinate individuals, exclusively during conspecific interactions. This modulation biases subordinates to engage in slower, non-LG-mediated, flexible escapes, while dominant individuals retain their ability to execute fast, LG-mediated escapes in response to unexpected attacks [114]. These findings suggest that neuromodulation might influence escape at multiple stages, from threat detection (see above) to escape execution.

Box 3 **Collective Escape Decisions**

Congregating with conspecifics can be an advantageous antipredation strategy. As the number of potential prey increases, the chance that a given individual will be predated decreases through dilution [145] and through the ‘confusion effect’ that hinders pursuit of single prey [146]. Importantly, grouping can increase the speed and accuracy of predator-avoidance decisions [147], and reduce the time devoted to threat vigilance through increased probability of detecting predators [148].

Foraging in a group can enhance threat detection in several ways. First, many animal species emit dedicated alarm signals to warn conspecifics of threatening situations. Various modalities of signals have been described, including chemical alarm signals [149] and alarm calls [150]. Alarm calls can carry information about the specific nature of a threat, such as the type and location of the detected predator [151], and can cause different responses in conspecifics: vervet monkeys tend to look up before initiating an escape following alarm calls elicited in response to eagles, whereas snake-elicited calls cause them to look down [152]. Emitting alarm calls when faced with predators may have the added benefit of accentuating dilution and confusion effects, but can also attract the attention of a predator [153]. It seems that animals can take this potential cost into consideration because they do not always emit alarm calls when faced with threat. Instead, sudden silence from conspecifics ceasing movement can function as a defence-inducing cue in rats [154], whereas crested pigeons preferentially flee from the sounds of escape take-offs of conspecifics versus routine take-offs [155]. In conditions of reduced visibility, where the benefit of hearing an alarm call increases, starlings increase the frequency of calls, suggesting that there is a dynamic cost–benefit computation controlling alarm-call emission [156]. A second means for increasing threat detection in groups is to observe the initiation of defensive behaviour by other individuals in the group. In some species of sparrows that emit little information about detected threat, individuals that do not directly detect threat infer it through the temporal profile of departures of other individuals from the flock [157].

In addition to threat detection, being in a group setting also influences escape execution, which becomes extremely dynamic due to the additional navigation constraints imposed by the presence of nearby animals also trying to escape. Fish schools, which are thought to be primarily an antipredator adaptation, provide an excellent example of collective escape behaviour. Schools of sand eels can execute diverse coordinated escape actions such as split, join, or hourglass formation [158], and in herring the type of escape formation depends on the approach angle of the threat [159]. For groups faced with predator-related decisions, it is likely that increases in apparent cognitive performance with group size are due to multiple mechanisms acting simultaneously, such as swarm intelligence and pool-of-competence effects 147, 160, and that their relative contributions are context-dependent.

Alt-text: Box 3

## Concluding Remarks

Although escape behaviour may appear to be simple, there is overwhelming evidence at the behavioural level that much more is involved than simple feedforward sensorimotor transformations. For systems neuroscience, escape behaviour provides a powerful ethological paradigm for studying the neural basis of cognitive processes such as perceptual and value-based decision-making, or goal-directed actions. While escape is often perceived as a simple stimulus-reaction, the lack of apparent explicit deliberation should not be taken as an indication of a simple computational process. Escape might need to be implemented under strong timing constraints and favour short reaction times, but even very fast escape responses can integrate multiple variables such as spatial constraints of the environment and economic trade-offs. The difference with other actions might be that the results of the computations relevant for successful escape are cached and ready to use (pre-computed cognitive constructs) instead of being computed *de novo* on the spot. This raises interesting parallels with heuristics-based decision-making, which might rely on similar processes, and thus investigation of escape mechanisms might shed light on this important component of behaviour. Although the neurophysiological mechanisms of threat detection and escape initiation have been studied in detail in some species, we know very little about how cognitive control of escape is implemented at the mechanistic level (see Outstanding Questions). As new software tools for rigorous behavioural quantification in freely behaving animals become available 115, 116, and are paired with high-density recordings of neural activity across multiple brain areas, our understanding of the neural basis of natural behaviours will increase at a fast pace. Most escape circuits receive projections from numerous telencephalic areas, and we anticipate that exciting advances in the field will come from investigating the intersection between cortical and subcortical circuits. This research avenue will improve not only our understanding of neural mechanisms of cognitive control of escape but, in doing so, will also advance our understanding of cortical function and cognition in general.

Outstanding Questions How, and where in the brain, is the choice between escape and other defensive actions computed? Prey can exhibit multiple defensive behaviours that are flexibly selected as a function of environment and threat contingencies, but which neural circuits implement this action selection? Are models of action selection developed for learned actions valid for instinctive behaviours, or are there specialised ‘low-level’ modules for computing instinctive choice of defence actions? Do the basal ganglia play a role in escape behaviour? To what extent do the apparent defensive strategies rely on deliberative processes?

How does the mammalian brain coordinate complex escapes? Fleeing to shelter may require navigation through complex environments and negotiation of obstacles or multiple route options, but where are these computations made? Can they be independently implemented by subcortical structures, or do they require coordination with cortical circuits? Where are variables that matter for escaping successfully encoded in the brain, and where are they integrated into escape decisions? Learning of these variables, such as shelter location, can be an extremely fast process, but how is this implemented at the neuronal and synaptic level?

How are experience-dependent changes in escape behaviour achieved at a neuronal level? Are they implemented as ‘top-down’ cortical control over subcortical areas, or are the midbrain circuits underlying innate behaviours themselves plastic? Are changes such as threat habituation long-lasting or even permanent? How are prior beliefs and expectations about threat and escape encoded and updated, and how do they influence the core escape-circuit modules?

## Glossary

**C-start**: a type of very fast escape made by fish and amphibians that begins with the body forming a characteristic C-shaped bend.
**Defensive behaviour**: behavioural actions that aim to minimize the chances of being harmed. When faced with predatory threats, animals can engage in different defensive behaviours such as escape, freezing, attack, alarm vocalizations, or thanatosis (‘playing dead’). Which behaviours they execute, and in which sequence, depends on properties specific to the predated species and its predator, as well as on its current environment, internal state, and previous experience.
**Escape**: the act of avoiding harm by increasing the distance of an agent from the source of threat, such as a predator, and possibly finding refuge in a safe location.
**Escape vigour**: the intensity of the escape action, which can be inferred from the kinematics of the escape, such as the speed, frequency, or amplitude of movement.
**Instinctive (behaviour)**: a class of behaviours ‘in which the motor pattern is variable but with an end result that is predictable from acknowledgment of the species, without knowing the history of the individual animal’ [161]. These behaviours can be driven by internal or external triggers, independently of learning.
**Innately threatening stimuli**: sensory stimuli that are perceived as threatening without requiring prior exposure or a learning process for acquiring a negative value.
**Kairomone**: an interspecific chemical signal that benefits the receiver rather than the emitter.
**Looming (stimulus)**: a stimulus that represents an approaching object. Visual looming stimuli are most often a 2D representation of an object on a collision course. The perceived threat level or imminence can be experimentally varied by changing the time of collision, size, velocity, or contrast of the visual stimulus.
