# Supplementary material for: Cognitive Control of Escape Behaviour
Source: Trends Cogn Sci. 2019 Apr;23(4):334–48. doi: 10.1016/j.tics.2019.01.012 (PMC6438863; doi:10.1016/j.tics.2019.01.012)
Supplement: Supplementary file 1 [file mmc1.pdf]

'Cognitive control of escape behaviour' – Supplemental references

Dominic A. Evans, A. Vanessa Stempel, Ruben Vale, Tiago Branco

Listed below are additional references that supplement those already cited in the main text; references are grouped according to general topic areas covered in the review, as indicated by the headings. Reviews are marked with an asterisk.

Threat detection

Circuits specialised to detect kairomones in vertebrates and invertebrates

- Papes, F. et al. (2010) The Vomeronasal Organ Mediates Interspecies Defensive Behaviors through Detection of Protein Pheromone Homologs. Cell 141, 692–703
- Martinez, R.C. et al. (2011) Amygdalar roles during exposure to a live predator and to a predator-associated context. Neuroscience 172, 314–328
- Ferrero, D.M. et al. (2011) Detection and avoidance of a carnivore odor by prey. Proc. Natl. Acad. Sci. 108, 11235–11240
- Ebrahim, S.A.M. et al. (2015) Drosophila Avoids Parasitoids by Sensing Their Semiochemicals via a Dedicated Olfactory Circuit. PLoS Biol. 13, 1–18
- Carvalho, V.M.A. et al. (2015) Lack of spatial segregation in the representation of pheromones and kairomones in the mouse medial amygdala. Front. Neurosci. 9, 283
- Liu, Z. et al. (2018) Predator-secreted sulfolipids induce defensive responses in C. elegans. Nat. Commun. 9:1128

Collision-detecting circuits and looming-sensitive cells across species

- Schlotterer, G.R. (1977) Response of the locust descending movement detector neuron to rapidly approaching and withdrawing visual stimuli. Can. J. Zool. 55, 1372–1376
- King, J.G. et al. (1999) Selective, unilateral, reversible loss of behavioral responses to looming stimuli after injection of tetrodotoxin or cadmium chloride into the frog optic nerve. Brain Res. 841, 20–26
- Gabbiani, F. et al. (2002) Multiplicative computation in a visual neuron sensitive to looming. Nature 420, 320–324
- Gahtan, E. et al. (2002) Evidence for a Widespread Brain Stem Escape Network in Larval Zebrafish. J. Neurophysiol. 87, 608–614
- Wu, L.Q. et al. (2005) Tectal neurons signal impending collision of looming objects in the pigeon. Eur. J. Neurosci. 22, 2325–2331
- Fotowat, H. et al. (2009) A Novel Neuronal Pathway for Visually Guided Escape in Drosophila melanogaster. J. Neurophysiol. 102, 875–885
- Münch, T.A. et al. (2009) Approach sensitivity in the retina processed by a multifunctional neural circuit. Nat. Neurosci. 12, 1308–1316
- Cohen, J.D. and Castro-Alamancos, M.A. (2010) Neural Correlates of Active Avoidance Behavior in Superior Colliculus. J. Neurosci. 30, 8502–8511
- Billington, J. et al. (2010) Neural processing of imminent collision in humans. Proc. R. Soc. 278, 1476–1481.
- Nakagawa, H. and Hongjian, K. (2011) Collision-Sensitive Neurons in the Optic Tectum of the Bullfrog, Rana catesbeiana. J. Neurophysiol. 104, 2487–2499
- Carbone, J. et al. (2018) Characterization and modelling of looming-sensitive neurons in the crab Neohelice. J. Comp. Physiol. 204, 487–503
- Dewell, R.B. and Gabbiani, F. (2018) Biophysics of object segmentation in a collision-detecting neuron. eLife. 7, e34238
- Dewell, R.B. and Gabbiani, F. (2018) M-current regulates firing mode and spike reliability in a collision-detecting neuron. J. Neurophysiol. 120, 1753-1764

Neural activity manipulations that elicit or modify defensive behaviours

- Hunsperger, R.W. (1956) Role of substantia grisea centralis mesencephali in electrically-induced rage reactions. Prog. Neurobiol. 2, 289-294
- Olds, M. and Olds, J. (1962) Approach-escape interactions in rat brain. Am. J. Physiol. 203, 803–810
- Halpern, M. (1968) Effects of midbrain central gray matter lesions on escape-avoidance behavior in rats. Physiol. Behav. 3, 171–178
- Nashold, B.J. et al. (1969) Sensations evoked by stimulation in the midbrain of man. J. Neurosurg. 30, 14–24
- Cools, A.R. et al. (1983) Picrotoxin microinjections into the brain: a model of abrupt withdrawal "jumping" behaviour in rats not exposed to any opiate? Eur. J. Pharmacol. 90, 237–243
- Cools, A.R. et al. (1984) The striato-nigro-collicular pathway and explosive running behaviour: functional interaction between neostriatal dopamine and collicular GABA. Eur. J. Pharmacol. 100, 71–77
- Sahibzada, N. et al. (1986) Movements Resembling Orientation or Avoidance Elicited by Electrical Stimulation of the Superior Colliculus in Rats. J. Neurosci. 6, 723–733

- Dean, P. et al. (1988) Responses resembling defensive behaviour produced by microinjection of glutamate into superior colliculus of rats. Neuroscience 24, 501–510
- Bandler, R. and Carrive, P. (1988) Integrated defence reaction elicited by excitatory amino acid microinjection in the midbrain periaqueductal grey region of the unrestrained cat. Brain Res. 439, 95–106.
- Northmore, D.P.M. et al. (1988) Behavior evoked by electrical stimulation of the hamster superior colliculus. Exp. Brain Res. 73, 595–605
- Sudré, E.C.M. et al. (1993) Thresholds of electrically induced defence reaction of the rat: Short- and long-term adaptation mechanisms. Behav. Brain Res. 58, 141–154
- Behbehani, M.M. (1995) Functional characteristics of the midbrain periaqueductal gray. Prog. Neurobiol. 46, 575–605
- De Oca, B.M. et al. (1998) Distinct regions of the periaqueductal gray are involved in the acquisition and expression of defensive responses. J. Neurosci. 18, 3426–3432
- Vianna, D.M.L. et al. (2001) Lesion of the Ventral Periaqueductal Gray Reduces Conditioned Fear but Does Not Change Freezing Induced by Stimulation of the Dorsal Periaqueductal Gray. Learn. Mem. 8, 164-169
- DesJardin, J.T. et al. (2013) Defense-Like Behaviors Evoked by Pharmacological Disinhibition of the Superior Colliculus in the Primate. J. Neurosci. 33, 150–155
- Liang, F. et al. (2015) Sensory Cortical Control of a Visually Induced Arrest Behavior via Corticotectal Projections. Neuron 86, 755–767
- Assareh, N. et al. (2016) The Organization of Defensive Behavior Elicited by Optogenetic Excitation of Rat Lateral or Ventrolateral Periaqueductal Gray. Behav. Neurosci. 130, 406-414

Previous predator encounters modify threat perception and escape

Behavioural data

- Melzack, R. (1961) On the Survival of Mallard Ducks after “Habituation” to the Hawk-Shaped Figure. Behaviour 17, 9–16
- Dill, L.M. (1974) The escape response of the zebra danio (Brachydanio rerio) II. The effect of experience. Anim. Behav. 22, 723–730
- Engel, J.E. and Hoy, R.R. (1999) Experience-dependent modification of ultrasound auditory processing in a cricket escape response. J. Exp. Biol. 202, 2797–2806
- Hazlett, B.A. (2003) Predator Recognition and Learned Irrelevance in the Crayfish Orconectes virilis. Ethology 109, 765–780
- Ferrari, M.C.O. et al. (2005) The role of learning in the development of threat-sensitive predator avoidance by fathead minnows. Anim. Behav. 70, 777–784
- Crook, R.J. et al. (2011) Peripheral injury induces long-term sensitization of defensive responses to visual and tactile stimuli in the squid Loligo pealeii, Lesueur 1821. J. Exp. Biol. 214, 3173–3185
- Crook, R.J. et al. (2014) Nociceptive sensitization reduces predation risk. Curr. Biol. 24, 1121–1125
- Chivers, D.P. et al. (2014) Background level of risk determines how prey categorize predators and non-predators. Proc. R. Soc. B Biol. Sci. 281, 20140355.
- Hegab, I.M. et al. (2014) The ethological relevance of predator odors to induce changes in prey species. Acta Ethol. 18, 1–9
- Brown, G.E. et al. (2015) Background risk and recent experience influences retention of neophobic responses to predators. Behav. Ecol. Sociobiol. 69, 737–745
- Collier, A. and Hodgson, J.Y.S. (2017) A Shift in Escape Strategy by Grasshopper Prey in Response to Repeated Pursuit. Southeast. Nat. 16, 503–515
- Nordell, C.J. et al. (2017) Flight initiation by Ferruginous Hawks depends on disturbance type, experience, and the anthropogenic landscape. PLoS One 12, 1–17
- Seehafer, K. et al. (2018) Ontogenetic and experience-dependent changes in defensive behavior in captive-bred Hawaiian bobtail squid, Euprymna scolopes. Front. Physiol. 9, 1–10
- Park, C. et al. (2018) Effects of Social Experience on the Habituation Rate of Zebrafish Startle Escape Response: Empirical and Computational Analyses. Front. Neural Circuits 12, 1–16
- Fanselow, M.S. (2018) The role of learning in threat imminence and defensive behaviors. Curr. Opin. Behav. Sci. 24, 44–49 *

Neural correlates and mechanisms

- Holmqvist, M.H. and Srinivasan, M. V. (1991) A visually evoked escape response of the housefly. J. Comp. Physiol. A 169, 451–459
- Engel, J.E. and Hoy, R.R. (1999) Experience-dependent modification of ultrasound auditory processing in a cricket escape response. J. Exp. Biol. 202, 2797–2806
- Yamamoto, K. et al. (2003) Input and Output Characteristics of Collision Avoidance Behavior in the Frog Rana catesbeiana. Brain. Behav. Evol. 62, 201–211
- Matheson, T. (2003) Plasticity in the Visual System Is Correlated With a Change in Lifestyle of Solitarious and Gregarious Locusts. J. Neurophysiol. 91, 1–12
- Gray, J.R. (2005) Habituated visual neurons in locusts remain sensitive to novel looming objects. J. Exp. Biol. 208, 2515–2532
- Fotowat, H. et al. (2009) A Novel Neuronal Pathway for Visually Guided Escape in Drosophila melanogaster. J. Neurophysiol. 102, 875–885

- Whitaker, K.W. et al. (2011) Serotonergic modulation of startle-escape plasticity in an African cichlid fish: a single-cell molecular and physiological analysis of a vital neural circuit. J. Neurophysiol. 106, 127–137
- Berón De Astrada, M. et al. (2013) Behaviorally related neural plasticity in the arthropod optic lobes. Curr. Biol. 23, 1389–1398
- Roberts, A.C. et al. (2016) Long-term habituation of the C-start escape response in zebrafish larvae. Neurobiol. Learn. Mem. 134, 360–368
- Magani, F. et al. (2016) Predation risk modifies behaviour by shaping the response of identified brain neurons. J. Exp. Biol. 219, 1172–1177
- Sitaraman, D. et al. (2017) Discrete Serotonin Systems Mediate Memory Enhancement and Escape Latencies after Unpredicted Aversive Experience in Drosophila Place Memory. Front. Syst. Neurosci. 11, 1–11
- Tomsic, D. et al. (2017) The predator and prey behaviors of crabs: from ecology to neural adaptations. J. Exp. Biol. 220, 2318–2327 *
- Almada, R.C. et al. (2018) Stimulation of the Nigrotectal Pathway at the Level of the Superior Colliculus Reduces Threat Recognition and Causes a Shift From Avoidance to Approach Behavior. Front. Neural Circuits 12, 1–9
- Burgos, A. et al. (2018) Nociceptive interneurons control modular motor pathways to promote escape behavior in Drosophila. Elife 7, 1–28

Cortical and neuromodulatory input to the superior colliculus

- Fries, W. (1984) Cortical projections to the superior colliculus in the macaque monkey: A retrograde study using horseradish peroxidase. J. Comp. Neurol. 230, 55–76
- Ueda, S. et al. (1985) The organization of serotonin fibers in the mammalian superior colliculus. An immunohistochemical study. Anat Embryol 173, 13–21
- Beitz, A.J. et al. (1986) Differential Origin of Brainstem Serotoninergic Projections to the Midbrain Periaqueductal Gray and Superior Colliculus of the Rat. J. Comp. Neurol. 250, 498–509
- May, P.J. (2006) The mammalian superior colliculus: Laminar structure and connections. Prog. Brain Res. 151, 321–378
- Muthuraju, S. et al. (2016) Dopamine D2 receptors regulate unconditioned fear in deep layers of the superior colliculus and dorsal periaqueductal gray. Behav. Brain Res. 297, 116–123
- Savage, M.A. et al. (2017) Segregated fronto-cortical and midbrain connections in the mouse and their relation to approach and avoidance orienting behaviors. J. Comp. Neurol. 525, 1980–1999

Associative learning expands the ability to detect threats

- LeDoux, J.E. et al. (1988) Different projections of the central amygdaloid nucleus mediate autonomic and behavioral correlates of conditioned fear. J. Neurosci. 8, 2517–2529
- Griffin, A.S. et al. (2001) Learning specificity in acquired predator recognition. Anim. Behav. 62, 577–589
- LeDoux, J. (2003) The emotional brain, fear, and the amygdala. Cell. Mol. Neurobiol. 23, 727–38 *
- Gross, C.T. and Canteras, N.S. (2012) The many paths to fear. Nat. Rev. Neurosci. 13, 651–658 *
- Pellman, B.A. and Kim, J.J. (2016) What Can Ethobehavioral Studies Tell Us about the Brain's Fear System? Trends Neurosci. 39, 420–431 *
- Mitchell, M.D. et al. (2015) Learning to distinguish between predators and non-predators: Understanding the critical role of diet cues and predator odours in generalisation. Sci. Rep. 5, 1–10
- Tovote, P. et al. (2015) Neuronal circuits for fear and anxiety. Nat. Rev. Neurosci. 16, 317–331 *

Vigilance and active risk assessment enhance threat detection

- Elgar, M. (1989) Predator vigilance and group size in mammals and birds: a critical review of the empirical evidence. Biol. Rev. 64, 13–33 *
- Stankowich, T. and Blumstein, D.T. (2005) Fear in animals: A meta-analysis and review of risk assessment. Proc. R. Soc. B Biol. Sci. 272, 2627–2634 *
- Hemmi, J.M. and Pfeil, A. (2010) A multi-stage anti-predator response increases information on predation risk. J. Exp. Biol. 213, 1484–1489
- Somerville, L.H. et al. (2011) Human bed nucleus of the stria terminalis indexed hypervigilant threat monitoring. Biol. Psychiatry 68, 416–424
- Pays, O. et al. (2013) Foraging in groups allows collective predator detection in a mammal species without alarm calls. Behav. Ecol. 24, 1229–1236
- Lee, S. et al. (2013) Direct Look from a Predator Shortens the Risk-Assessment Time by Prey. PLoS One 8, e64977
- Chivers, D.P. et al. (2016) Risk assessment and predator learning in a changing world: Understanding the impacts of coral reef degradation. Sci. Rep. 6, 1–7
- McNaughton, N. and Corr, P.J. (2018) Survival circuits and risk assessment. Curr. Opin. Behav. Sci. 24, 14–20
- Lojowska, M. et al. (2018) Visuocortical changes during a freezing-like state in humans. Neuroimage 179, 313–325
- Blank, D.A. (2018) Escaping behavior in goitered gazelle. Behav. Processes 147, 38–47

Decision processes and economics control the onset of escape

- Cooper, W.E. and Frederick, W.G. (2007) Optimal flight initiation distance. J. Theor. Biol. 244, 59–67
- Samia, D.S.M. et al. (2016) Fifty years of chasing lizards: New insights advance optimal escape theory. Biol. Rev. 91, 349–366 *
- Blumstein, D.T. et al. (2016) Escape behavior: dynamic decisions and a growing consensus. Curr. Opin. Behav. Sci. 12, 24–29 *
- Cooper, W.E. (2016) Fleeing to refuge: Escape decisions in the race for life. J. Theor. Biol. 406, 129–136 *
- Tätte, K. et al. (2018) Towards an integrated view of escape decisions in birds: relation between flight initiation distance and distance fled. Anim. Behav. 136, 75–86

Behavioural state and social context control defensive behaviours

- Bellman, K.L. and Krasne, F.B. (1983) Adaptive complexity of interactions between feeding and escape in crayfish. Science. 221, 779–781
- Cooper, W.E. (1997) Factors Affecting Risk and Cost of Escape by the Broad-Headed Skink (Eumeces laticeps): Predator Speed, Directness of Approach, and Female Presence. Herpetologica 53, 464–474
- Díaz-Uriarte, R. (1999) Anti-predator behaviour changes following an aggressive encounter in the lizard Tropidurus hispidus. Proc. R. Soc. B Biol. Sci. 266, 2457–2464
- Lin, D. et al. (2011) Functional identification of an aggression locus in the mouse hypothalamus. Nature 470, 221–6
- Issa, F.A. et al. (2012) Neural Circuit Reconfiguration by Social Status. J. Neurosci. 32, 5638–5645
- Madden, J.R. and Whiteside, M.A. (2014) Selection on behavioural traits during ‘unselective’ harvesting means that shy pheasants better survive a hunting season. Anim. Behav. 87, 129–135
- Clinchy, M. et al. (2013) Predator-induced stress and the ecology of fear. Funct. Ecol. 27, 56–65
- Niemelä, P.T. et al. (2015) Personality-related survival and sampling bias in wild cricket nymphs. Behav. Ecol. 26, 936–946
- Falkner, A.L. et al. (2016) Hypothalamic control of male aggression-seeking behavior. Nat. Neurosci. 19, 596–604
- Blake, C.A. and Gabor, C.R. (2016) Exploratory behaviour and novel predator recognition: behavioural correlations across contexts. J. Fish Biol. 89, 1178–1189
- Livneh, Y. et al. (2017) Homeostatic circuits selectively gate food cue responses in insular cortex. Nature 546, 611
- Park, C. et al. (2018) Effects of Social Experience on the Habituation Rate of Zebrafish Startle Escape Response: Empirical and Computational Analyses. Front. Neural Circuits 12, 1–16

Freezing as a defence mechanism

- O’Brien, T.J. and Dunlap, W.P. (1975) Tonic immobility in the blue crab (Callinectes sapidus, Rathbun): Its relation to threat of predation. J. Comp. Physiol. Psychol. 89, 86–94
- Bonenfant, M. and Kramer, D.L. (1996) The influence of distance to burrow on flight initiation distance in the woodchuck, Marmota monax. Behav. Ecol. 7, 299–303
- Mongeau, R. et al. (2003) Neural correlates of competing fear behaviors evoked by an innately aversive stimulus. J. Neurosci. 23, 3855–3868
- Edut, S. and Eilam, D. (2004) Protean behavior under barn-owl attack: Voles alternate between freezing and fleeing and spiny mice flee in alternating patterns. Behav. Brain Res. 155, 207–216
- Eilam, D. (2005) Die hard: A blend of freezing and fleeing as a dynamic defense - implications for the control of defensive behavior. Neurosci. Biobehav. Rev. 29, 1181–1191 *
- Roelofs, K. (2017) Freeze for action: Neurobiological mechanisms in animal and human freezing. Philos. Trans. R. Soc. B Biol. Sci. 372, 20160206 *

Escape tactics

Optimising the speed of escape

- Walker, J.A. et al. (2005) Do faster starts increase the probability of evading predators? Funct. Ecol. 19, 808–815
- Seamone, S. et al. (2014) Sharks modulate their escape behavior in response to predator size, speed and approach orientation. Zoology 117, 377–382
- Wheatley, R. et al. (2015) How Fast Should an Animal Run When Escaping? An Optimality Model Based on the Trade-Off between Speed and Accuracy. Integr. Comp. Biol. 55, 1166–1175
- Nasir, A.F.A.A. et al. (2017) Optimal running speeds when there is a trade-off between speed and the probability of mistakes. Funct. Ecol. 31, 1941–1949
- Nair, A. et al. (2017) A faster escape does not enhance survival in zebrafish larvae. Proc. R. Soc. B Biol. Sci. 284, 20170359

Escape trajectories and tactics

- Arnott, S. et al. (1999) Escape trajectories of the brown shrimp crangon crangon, and a theoretical consideration of initial escape angles from predators. J. Exp. Biol. 202 (Pt 2), 193–209
- Lindeyer, C.M. and Reader, S.M. (2010) Social learning of escape routes in zebrafish and the stability of behavioural traditions. Anim. Behav. 79, 827–834
- Mäkeläinen, S. et al. (2014) Different escape tactics of two vole species affect the success of the hunting predator, the least weasel. Behav. Ecol. Sociobiol. 68, 31–40
- Cooper, W.E. and Sherbrooke, W.C. (2016) Strategic Escape Direction: Orientation, Turning, and Escape Trajectories of Zebra-Tailed Lizards (Callisaurus draconoides). Ethology 122, 542–551
- Herbert-Read, J.E. et al. (2017) Escape path complexity and its context dependency in Pacific blue-eyes (Pseudomugil signifer). J. Exp. Biol. 220, 2076–2081
- Fukutomi, M. and Ogawa, H. (2017) Crickets alter wind-elicited escape strategies depending on acoustic context. Sci. Rep. 7, 1–8

Neuroethology of escape and decision-making: reviews

- Adams, G.K. et al. (2012) Neuroethology of decision-making. Curr. Opin. Neurobiol. 22, 982–989
- Blumstein, D.T. et al. (2016) Escape behavior: dynamic decisions and a growing consensus. Curr. Opin. Behav. Sci. 12, 24–29
- Korn, H. and Faber, D.S. (2005) The Mauthner cell half a century later: A neurobiological model for decision-making? Neuron 47, 13–28
- Lagos, P.A. (2017) A review of escape behaviour in orthopterans. J. Zool. 303, 165–177
- Ledoux, J. and Daw, N.D. (2018) Surviving threats: Neural circuit and computational implications of a new taxonomy of defensive behaviour. Nat. Rev. Neurosci. 19, 269–282
- McNaughton, N. and Corr, P.J. (2018) Survival circuits and risk assessment. Curr. Opin. Behav. Sci. 24, 14–20
- Mobbs, D. (2018) The ethological deconstruction of fear(s). Curr. Opin. Behav. Sci. 24, 32–37
